# Early and long-term prognosis in patients with and without type 2 diabetes after carotid intervention: a Swedish nationwide propensity score matched cohort study

**DOI:** 10.1186/s12933-021-01282-x

**Published:** 2021-04-24

**Authors:** Alexander Zabala, Anders Gottsäter, Marcus Lind, Ann-Marie Svensson, Björn Eliasson, Rebecka Bertilsson, Jan Ekelund, Thomas Nyström, Magnus Jonsson

**Affiliations:** 1 Department of Clinical Science and Education, Karolinska Institutet, Södersjukhuset, 11883 Stockholm, Sweden; 2 Department of Clinical Sciences, Malmö, Lund University, Lund, Sweden; 3 Vascular Center, Department of Cardio Thoracic Surgery and Vascular Diseases, Skåne University Hospital, Malmö, Sweden; 4 Department of Molecular and Clinical Medicine, Institute of Medicine, University of Gothenburg, Gothenburg, Sweden; 5 Department of Medicine, NU Hospital Group, Uddevalla, Sweden; 6 Centre of Registers in Region Västra Götaland, Gothenburg, Sweden; 7 Institute of Medicine, University of Gothenburg, Gothenburg, Sweden; 8 Department of Molecular Medicine and Surgery, Karolinska Institutet, Stockholm, Sweden; 9 Department of Vascular Surgery, Karolinska University Hospital, Stockholm, Sweden

**Keywords:** Carotid stenosis, Carotid artery stenting, Carotid endarterectomy, Stroke, Type 2 diabetes

## Abstract

**Objectives:**

To investigate early and long-term outcomes after treatment of carotid artery stenosis in patients with type 2 diabetes (T2D) compared to patients without T2D.

**Design/method:**

This observational nationwide population-based retrospective cohort study investigated all T2D patients treated for carotid stenosis registered in the National Swedish Vascular Surgery and the National Diabetes Registries. Data was collected prospectively for all patients after carotid intervention, during 2009–2015. We estimated crude early (within 30-days) hazard ratios (HRs) risk of stroke and death, and long-term HRs risk, adjusted for confounders with 95% confidence intervals (CIs), for stroke and death and major adverse cardiovascular events (MACE) by using inverse probability of treatment weighting matching.

**Results:**

A total of 1341 patients with T2D and 4162 patients without T2D were included; 89% treated for symptomatic carotid stenosis, 96% with carotid endarterectomy. There was an increased early risk, HRs (95% CI), for stroke in T2D patients 1.65 (1.17–2.32), whereas risk for early death 1.00 (0.49–2.04) was similar in both groups. During a median follow-up of 4.3 (T2D) and 4.6 (without T2D), with a maximum of 8.0 years; after propensity score matching there was an increased HRs (95% CI) of stroke 1.27 (1.05–1.54) and death 1.27 (1.10–1.47) in T2D patients compared to patients without T2D. Corresponding numbers for MACE were 1.21 (1.08–1.35).

**Conclusions:**

Patients with T2D run an increased risk for stroke, death, and MACE after carotid intervention. They also have an increased perioperative risk for stroke, but not for death.

**Supplementary Information:**

The online version contains supplementary material available at 10.1186/s12933-021-01282-x.

## Introduction

The prevalence of diabetes has increased dramatically the past decades all over the world, and type 2 diabetes (T2D) accounts for the majority of the cases. T2D has been associated with an increased risk of cardiovascular complications [1]. People with T2D have more than a 50% relative higher risk for stroke compared to those without diabetes [2].

Stroke represents a heterogeneous group of vascular pathologies where risk factors, such as hypertension, atrial fibrillation, smoking, hyperlipidemia and carotid stenosis, contribute to the increased risk. Carotid stenosis accounts for approximately 10–15% of all ischemic strokes [3]. Ever since the landmark studies, i.e. the North American Symptomatic Carotid Endarterectomy Trial (NASCET) [4] and the European Carotid Surgery Trial (ECST) [5], carotid endarterectomy has been a cornerstone in reducing the risk of stroke in patients with symptomatic carotid stenosis.

Individuals with diabetes have an increased risk of mortality after surgical revascularization of the coronary arteries [4] demonstrated by several trials [5]. Today no trial has investigated the risk of stroke and death in patients with diabetes compared to patients without diabetes, after carotid intervention. Also, large observational studies about the relationship between diabetes and long-term outcome of stroke and death after carotid intervention are scarce [6]. Since a large proportion of patients undergoing surgery for carotid stenosis have T2D, it is of importance to investigate and evaluate cardiovascular outcomes after such procedures in those patients.

Therefore, we wanted to investigate early risk of stroke and death, and long-term prognosis of stroke, death, and major adverse cardiovascular events (MACE) after carotid intervention in T2D patients compared to patients without T2D, in a nationwide observational cohort study.

## Methods

This observational population-based retrospective cohort study investigated all patients who underwent a surgical procedure for carotid stenosis registered in the Swedish National Registry for Vascular Surgery (Swedvasc). Data was collected prospectively for all patients with symptomatic or asymptomatic carotid stenosis treated with carotid endarterectomy (CEA) or carotid artery stenting (CAS) during 2009 to 2015.

### Databases and definitions

The unique personal identity number assigned to every Swedish citizen was used as the identifier in the records linkage procedure at the Swedish National Board of Health and Welfare. The database was then anonymized according to regulations. Each of the following registers were linked to the Swedvasc register, i.e. the Swedish National Diabetes Register (NDR), the Swedish National Patient Register (NPR), the Swedish Cause of Death Register, the Swedish Prescribed Drug Register (PDR) and the Longitudinal Integration Database for Health Insurance and Job Market Studies Register (LISA) [7].

All patients undergoing vascular surgery in Sweden are registered in Swedvasc, which is a national patient register established in 1994. Type of intervention, i.e. CEA and CAS, together with preoperative data such as: risk factors, type of treatment, complications and reinterventions are registered in Swedvasc and followed up to 30-days. The external validation of carotid interventions in Swedvasc is almost 100% [8].

The NDR includes more than 94% of all patients ≥ 18 years diagnosed with T2D in Sweden. Healthcare providers report continuously directly to the NDR or via electronic patient records from routine clinical practice. The registry includes data on clinical characteristics, diabetes treatment, risk factors, and diabetes-related complications. T2D was defined according to epidemiological criteria: that is, treatment with oral glucose-lowering treatment combined with diet, or diet only; or individuals aged ≥ 40 years at the time of diagnosis treated with glucose-lowering treatment combined with insulin, or insulin only [9].

In this study, all patients registered in Swedvasc after surgery for carotid stenosis between 2009 and 2015 were identified. Symptomatic carotid stenosis was defined as an ipsilateral acute neurologic event, i.e. stroke or transient ischemic attack within 180 days caused by a carotid stenosis. Asymptomatic carotid stenosis was defined as a carotid stenosis without a prior neurologic event, or neurologic event more than 180-days ago. All registered patients with a defined carotid stenosis were then associated with the registration in NDR and defined as T2D patients and compared with those without T2D diabetes. Study reporting follows the STROBE guidelines for observational studies using routinely collected data [10].

### Baseline demographic data

Demographic characteristics were obtained from Swedvasc, however when data was missing in Swedvasc, NDR was used to complement. Information about preexisting history of comorbidities were retrieved from IPR and NDR including: cardiovascular disease, stroke, myocardial infarction, coronary heart disease, heart failure, atrial fibrillation, kidney disease, cancer disease, gastric bypass, psychiatric disorder, and dementia.

Use of pharmacology drug treatment such as: anticoagulation therapy, acetylsalicylic acid, anti-hypertensive, lipid-lowering, and glucose-lowering treatment, were retrieved from PDR, and was defined as three filled, prescriptions for medication for 1 year prior to index date, to indicate actual usage.

The LISA register was used to obtain socioeconomic information such as: educational level, marital status, household disposable income and country of birth.

### Outcome and follow-up

Since Swedvasc only provides 30-days outcome after the procedure, the IPR was used to evaluate long-term outcomes. The study population was followed from first carotid interventional procedure (defining the index date) until outcome in the form of: stroke, death, cardiovascular death, MACE, i.e. non-fatal MI, non-fatal stroke and cardiovascular death, or end of study, i.e., December 31, 2017. Only the first hospital admission was used for patients with several of the outcome episodes.

The Cause of Death Register was used to obtain the dates and causes of death. For the cardiovascular death outcome, the following ICD 10 codes were used: I20–I25, I61–I64, R57.0 and R96. ICD 10 codes were used to identify all strokes: I63 and I64 were used for ischemic stroke, I61 to I62 were used for hemorrhagic stroke. Subarachnoid hemorrhage (ICD 10 code I60) was not included. Myocardial infarction was defined by ICD 10 code I21, and heart failure by ICD 10 code I50.

Only the first hospital admission was used for participants with several stroke episodes. Since Swedvasc is a code-based register, information on stroke laterality was not possible to determine. The validity of the diagnoses of stroke, myocardial infarction and heart failure in NPR has previously been evaluated with positive predictive values between 85 and 95% [11].

### Statistical analysis

Descriptive statistics are presented using mean, median, standard deviation (SD), interquartile range (IQR), counts, and percentages with 95% confidence intervals (CI) according to variable type. The degree of similarity between T2D patients and patients without T2D is described using the standardized mean difference. Cumulative incidences of all-cause mortality, cardiovascular death, stroke, and MACE are described using Kaplan–Meier curves with lifetables transformed to estimate distribution function rather than survival function. P-values < 0.05 were considered significant.

Outcomes were compared between patients with and without T2D at the end of follow-up. Due to length of follow-up varying across patients a Cox proportional hazards ratios (HRs) model was used to compare groups using both unadjusted Cox-regression and Inverse Probability of Treatment Weighting (IPTW), i.e., propensity score matching, adjusted Cox-regression and results are presented as hazard ratios (HRs) with associated 95% CI. Propensity scores were estimated adjusted for the following variables; age, sex, smoking, indication (asymptomatic/symptomatic), type of intervention (CEA/CAS), lipid lowering treatment, antihypertensive treatment, acetylsalicylic acid, clopidogrel, other oral anticoagulant, ACE-inhibitors, angiotensin receptor blockers, beta blocker, calcium channel blocker, disposable income, education, marital status, country of origin, acute myocardial infarct, coronary heart disease, stroke, cardiovascular disease, atrial fibrillation, heart failure, renal disease, cancer, psychiatric disorder, coronary revascularisation, dementia, gastric bypass, and peripheral arterial disease (Additional file 1: Table S1). When estimating propensity score for comparisons within sub-groups, indication and type of surgery were excluded as appropriate. Propensity scores were estimated using a generalized boosted multinomial regression model with an interaction depth of three, a maximum of 10,000 trees, and a shrinkage of 0.01. The optimal number of trees were selected using a stopping rule applied to the degree of balance.

Most variables were derived from mandatory health data or population registries and therefore have virtually no missing values, except smoking for which 16% of data was missing. The gradient boosting machine model used in the estimation of the propensity score treats missing values as a separate category and attempts to balance the proportion of missing values as well as non-missing values. Analyses were performed using R 3.4.3.

## Results

### Study population and demographic characteristics

A total of 5503 patients were registered in Swedvasc during the period Jan 1st, 2009–Dec 31st, 2015 with a carotid intervention. Out of these 5503 patients 1341 patients (24.4%) had T2D. The majority of the patients were treated for symptomatic CAS (88.6% patients without T2D vs. 89% T2D patients) and CEA was the most common intervention (95.9% patients without T2D vs. 94.6% T2D patients).

All baseline demographic characteristics of the patients studied are presented in Table 1. Mean age was 72 (SD 8) years in both groups, with a larger proportion of females among patients without T2D (33.0%) compared to T2D patients (28.2%). The majority of the patients were treated for a high-grade stenosis (70–99% NASCET), a small proportion of patients with repeated symptoms were treated for a stenosis of less than 50%, 6% of the patients had a contralateral stenosis in both groups. Patients without T2D were more likely to smoke compared to T2D patients (25.8% vs. 20.1%). In contrast, it was more common that T2D patients had a history of cardiovascular comorbidities compared to patients without T2D (Table 1).

**Table 1:** Baseline characteristics of individuals [without 2 diabetes (non-T2D) vs. type 2 diabetes (T2D)] undergoing carotid intervention

	Non-T2D	T2D	P-value	SMD
n	4162	1341		
Age, mean (SD)	72.21 (8.30)	72.42 (7.72)	0.416	0.026
Female, n (%)	1372 (33.0)	378 (28.2)	0.010	0.104
Smoking, n (%)	875 (25.8)	248 (20.1)	< 0.001	0.134
History of comorbidities, n (%)				
Cardiovascular disease (%)	2265 (54.4)	851 (63.5)	< 0.001	0.185
Stroke	1989 (47.8)	743 (55.4)	< 0.001	0.153
Myocardial infarction	483 (11.6)	243 (18.1)	< 0.001	0.184
Coronary heart disease	1119 (26.9)	539 (40.2)	< 0.001	0.285
Heart failure	243 (5.8)	138 (10.3)	< 0.001	0.164
Atrial fibrillation	460 (11.1)	216 (16.1)	< 0.001	0.148
Kidney disease	150 (3.6)	86 (6.4)	< 0.001	0.129
Cancer disease	450 (10.8)	137 (10.2)	0.573	0.019
Gastric bypass	2 (0.0)	2 (0.1)	0.541	0.032
Psychiatric disorder	156 (3.7)	41 (3.1)	0.272	0.038
Dementia	17 (0.4)	5 (0.4)	1.00	0.006
Degree of ipsilateral carotid stenosis, n (%)^a^			0.52	0.036
≤ 50	229 (5.5)	82 (6.1)		
50–69	1194 (28.7)	397 (29.6)		
70–99	2737 (65.8)	862 (64.3)		
Degree of contralateral carotid stenosis, n (%)^a^			0.19	0.068
≤ 50	3105 (74.7)	966 (72.0)		
50–69	454 (10.9)	163 (12.2)		
70–99	349 (8.4)	132 (9.8)		
Occlusion	251 (6.0)	80 (6.0)		
Symptomatic stenosis, n (%)	3686 (88.6)	1194 (89.0)	0.669	0.015
Carotid endarterectomy, n (%)	3993 (95.9)	1269 (94.6)	0.050	0.062
Peripheral arterial disease (%)	175 (4.2)	101 (7.5)	< 0.001	0.142
Medication, n (%)				
Lipid lowering drug	2623 (63.0)	1061 (79.1)	< 0.001	0.361
Antihypertensive drug	3192 (76.7)	1222 (91.1)	< 0.001	0.401
ACE inhibitor	1154 (27.7)	533 (39.7)	< 0.001	0.256
Angiotensin II receptor blocker	755 (18.1)	331 (24.7)	< 0.001	0.160
Beta blocker	1740 (41.8)	777 (57.9)	< 0.001	0.327
Calcium chanel blocker	1379 (33.1)	640 (47.7)	< 0.001	0.301
Anticoagulant therapy^b^	1434 (34.5)	542 (40.4)	< 0.001	0.123
Acetylsalicylic acid	2517 (60.5)	897 (66.9)	< 0.001	0.134
P2Y12 inhibitor (Clopidogrel)	757 (18.2)	293 (21.8)	0.003	0.092
Antihyperglycaemic agent, n (%)				
Metformin	0 (0.0)	744 (55.5)	< 0.001	1.579
Sodium–glucose-transport-2 inhibitor	0 (0.0)	4 (0.3)	< 0.001	0.077
Incretin^c^	0 (0.0)	74 (5.5)	< 0.001	0.342
Insulin	0 (0.0)	492 (36.7)	< 0.001	1.077
Disposable income per month after tax, USD	2078.3 (2609)	1875.8 (204)	0.009	0.086
Educational level, n (%)			0.012	0.096
Compulsory school	1658 (40.3)	567 (42.8)		
Upper secondary	1707 (41.5)	562 (42.4)		
College/university	753 (18.3)	196 (14.8)		
Civil status, n (%)			0.092	0.079
Single	385 (9.3)	132 (9.9)		
Married	2294 (55.2)	712 (53.1)		
Divorced	822 (19.8)	303 (22.6)		
Widowed	657 (15.8)	193 (14.4)		
Origin, n (%)			0.015	0.088
Sweden	3574 (85.9)	1112 (82.9)		
Europe except Sweden	298 (7.2)	106 (7.9)		
Rest of the world	290 (7.0)	123 (9.2)		

Categorical variables are presented as number (%) and continuous variables are presented as mean (SD)

*SMD* standardised mean difference, *SD* standard deviation

^a^Definition accordingly to The North American Symptomatic Carotid Endarterectomy Trial

^b^Anticoagulant therapy includes heparin, low molecular heparin, non-vitamin K antagonist and vitamin K antagonists

^c^Incretin, includes dipeptidyl peptidase-4 inhibitors and glucagon-like peptide-1

Lipid lowering drugs were used by 79.1% of the T2D patients, and by 63.0% among patients without T2D. Also, antiplatelet drugs were more frequently used in T2D patients; acetylsalicylic acid 66.9% vs. 60.5%, and clopidogrel 21.8% vs. 18.2%, respectively. Patients without T2D had a higher education level and an increased socioeconomic level than T2D patients (Table 1). There were no differences in the degree of ipsilateral or contralateral degree of carotid stenosis between groups (Table 1). Glycemic control and diabetes duration in T2D patients were HbA1c 55.6 (SD 14.2) mmol/mol (HbA1c 7.2%) and 10.5 (SD 7.9) years, respectively.

### Early (within 30-days) complication rates

The perioperative (within 30-days) complication rate for stroke was 3.7% (95% CI 2.7–4.7%) compared to 2.3% (95% CI 1.8–2.7%) for patients without T2D. There was no difference in rates of perioperative death between groups: T2D patients 0.7% (95% CI 0.5–1.0%) vs. patients without T2D 0.7% (95% CI 0.3–1.2%), respectively. Corresponding early crude HRs (95% CI) for stroke was 1.65 (1.17–2.32) in T2D patients compared to patients without T2D, whereas early crude risk for death was similar 1.00 (0.49–2.04) in both groups.

### Long-term outcome events and crude incidence rate between groups after carotid intervention

**Fig. 1:**
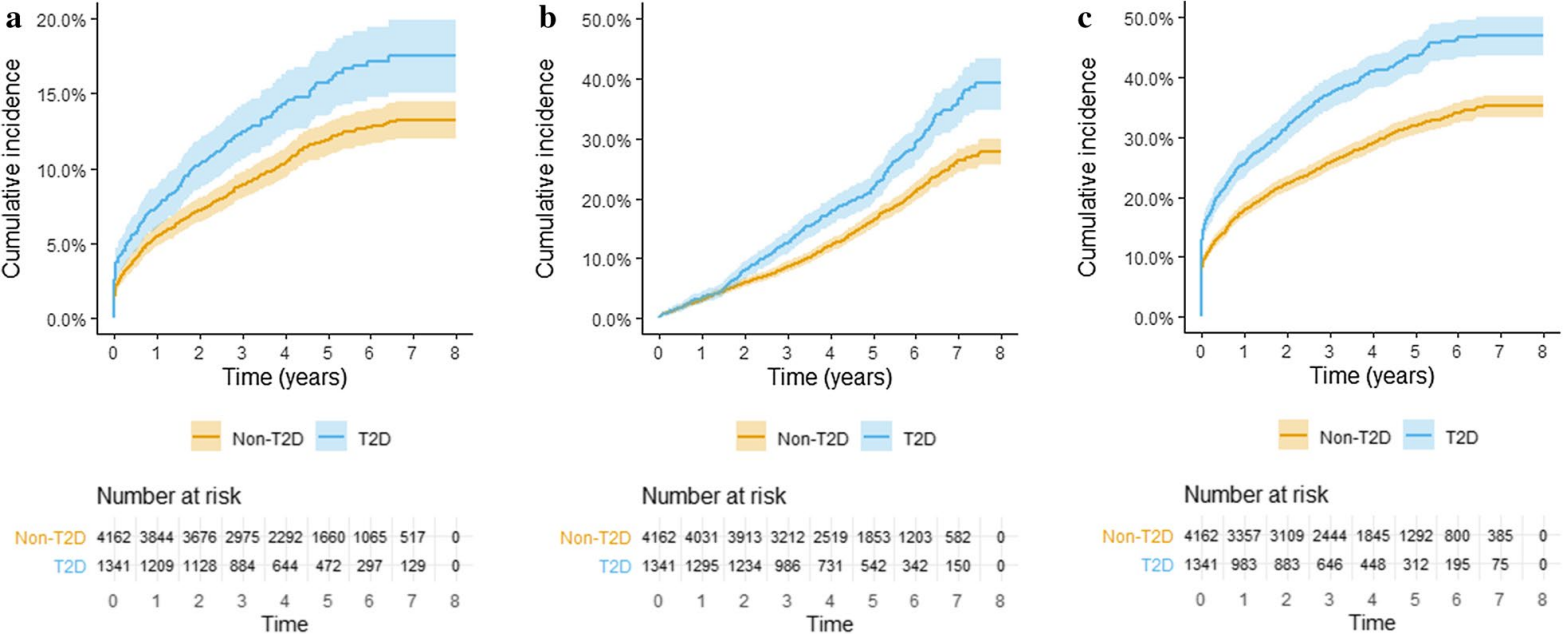
Crude Kaplan–Meier curves demonstrating cumulative incidence and number at risk of **a** stroke **b** death, and **c** MACE (major adverse cardiovascular events, i.e. non-fatal myocardial infarction, non-fatal stroke and cardiovascular death) after carotid interventions (i.e., carotid endarterectomy and carotid artery stenting) among patients without type 2 diabetes (non-T2D) and patients with type 2 diabetes (T2D). Shaded areas represent 95% CI

Median follow up time 4.3 (IQR 2.9–6.0) years for T2D patients and 4.6 (IQR 3.1–6.3) years for patients without T2D with a maximum follow-up time of 8.0 years (both groups). Kaplan–Meier estimated survival curves demonstrating the cumulative number of patients at risk and the proportions for the outcomes, i.e., stroke, death, and MACE are shown in Fig. 1a–c. Numbers of event and crude incidence rate for the studied outcomes were all increased in T2D patients compared to patients without T2D (Table 2).

**Table 2:** Total number of events and incidence rate with exact 95% Poisson confidence interval for the outcomes of individuals without type 2 diabetes (Non-T2D) and individuals with type 2 diabetes (T2D), respectively, undergoing carotid intervention

Outcome	Non-T2D (n = 4162)		T2D (n = 1341)	
	Events (n)	Incidence rate (per 1000 patients)	Events (n)	Incidence rate (per 1000 patients)
Stroke	453	25.1 (22.8–27.5)	193	35.7 (30.8–41.1)
Ischemic stroke	380	20.8 (18.8–23.0)	166	30.3 (25.9–35.3)
Hemorrhagic stroke	68	3.5 (2.7–4.5)	20	3.4 (2.1–5.2)
Death	725	37.4 (34.7–40.2)	324	54.5 (48.7–60.7)
Cardiovascular death	161	8.3 (7.1–9.7)	89	15.0 (12.0–18.4)
MACE	1248	82.7 (78.2–87.4)	551	135 (123.4–146.1)

*HR* hazard ratio, *MACE* major adverse cardiovascular events, i.e. non-fatal myocardial infarction, non-fatal stroke and cardiovascular death

### Propensity score matched risk of long-term outcomes between groups after carotid intervention

After propensity score matching the groups were well balanced concerning all covariates and demographic characteristics (Additional file 1: Table S2). HRs (95% CI) for stroke, death, cardiovascular death, and MACE were all increased in patients with T2D: 1.27 (1.05–1.54), 1.27 (1.10–1.47), 1.60 (1.20–2.12), and 1.21 (1.08–1.35), respectively, compared to patients without T2D (Table 3). The corresponding numbers of the HRs for the outcomes, after dividing groups in symptomatic vs. asymptomatic carotid stenosis, and CEA vs. CAS procedures, are shown in Additional file 1: Tables S3 and S4, respectively. The proportion of events for CAS and asymptomatic stenosis compared to CEA and symptomatic stenosis were small (Additional file 1: Tables S3 and S4).

**Table 3:** Cox-regression estimates [crude and after adjustments using inverse probability of treatment weighting (IPTW)] for individuals with type 2 diabetes compared with individuals without type 2 diabetes with asymptomatic and symptomatic carotid stenosis undergoing any carotid intervention

Outcome	Crude HR (95% CI)	P-value	IPTW adjustment^a^ HR (95% CI)	P-value
Stroke	1.38 (1.17–1.64)	< 0.001	1.27 (1.05–1.54)	0.012
Ischemic stroke	1.41 (1.18–1.69)	< 0.001	1.30 (1.06–1.60)	0.011
Hemorrhagic stroke	0.95 (0.58–1.56)	0.834	1.29 (1.06–1.55)	0.009
Death	1.48 (1.30–1.68)	< 0.001	1.27 (1.10–1.47)	0.001
Cardiovascular death	1.79 (1.38–2.32)	< 0.001	1.60 (1.20–2.12)	0.001
MACE	1.52 (1.37–1.68)	< 0.001	1.21 (1.08–1.35)	0.001

*CI* confidence interval, *HR* hazard ratio, *MACE* major adverse cardiovascular events. i.e. non-fatal myocardial infarction, non-fatal stroke and cardiovascular death, *IPTW* inverse probability of treatment weighting

^a^Adjusted for all variables in Additional file 1: Table S1

## Discussion

In this large nationwide cohort of patients that underwent carotid surgery in Sweden during a follow-up period of 8 years, we observed that patients with T2D had an increased risk of stroke and death compared to patients without T2D after carotid intervention. Perioperatively there was an increased risk of stroke, but not for death, in T2D patients.

In previous studies the difference in perioperative complications between patients with or without diabetes has mainly been attributed to mortality [6, 12]. This is in contrast with the current observational study in which rates of perioperative death were similar between groups after carotid intervention. Even though there was an increased perioperatively risk of stroke for T2D patients, the risk was below the recommended threshold of 6% to justify carotid intervention [13].

In the present study long-term risk of stroke, death, and cardiovascular events (MACE) were all increased in T2D patients compared to patients without T2D. This contradicts a recent observational study that demonstrated increased perioperative mortality in the presence of diabetes after carotid surgery, a difference that was negligible during follow-up [6]. Other studies report different results regarding prognosis; some have reported that patients with diabetes are not at an increased risk [6], while others support the role of diabetes as an increased risk factor for stroke and death after carotid intervention [13]. Increased long-term mortality after carotid intervention in diabetic patients compared to patients without diabetes was recently demonstrated in a meta-analysis comprising more than 4000 patients with diabetes [14]. However, since a minority of the studies included in the meta-analysis only reported mortality; long-term prognosis of stroke or cardiovascular events after carotid intervention in patients with diabetes is still to a large extent unknown [14].

### Cardiovascular risk factors and diabetes

Diabetes is a well-known risk factor for adverse cardiovascular events, and must be paid attention to, when choosing type of revascularization in multivessel coronary artery disease as certain procedures, i.e. coronary artery bypass grafting, is superior to others [15]. Patients with diabetes have an accelerated and more extensive coronary atherosclerosis than patients without diabetes [16]. In carotid arteries, studies demonstrating signs of pronounced atherosclerosis in patients with diabetes compared to patients without diabetes [17]. Already in children with T2D, carotid intima-media thickness is increased [18], which in turn is associated with future cardiovascular events [19]. Strong cardiovascular risk factors such as smoking, hypertension, and dyslipidaemia are well established contributing risk factors for carotid stenosis. In insulin resistance, more or less obligate in T2D patients, some strong cardiovascular risk factors are concomitantly represented, i.e., hypertension, obesity, dyslipidaemia, kidney failure, and dysglycemia [20], which all can have contributed to the increased risk of stroke and death and MACE after carotid surgery observed in the current study. Interestingly, insulin resistance per se is associated with increased intima media thickness in diabetes [9]. In the present study, only smoking status was collected in the Swedvasc register and subsequently adjusted for between groups, whereas the other above mentioned metabolic cardiovascular risk factors were not compared between groups which may all have contributed to the increased cardiovascular risk after carotid intervention observed in T2D patients.

Not only metabolic factors can contribute to an increased risk of cardiovascular events after carotid intervention, co-morbidities and medications for reducing cardiovascular risk are also relevant. In the present study, at baseline cardiovascular comorbidities and medications such as lipid-lowering and anti-platelets agents were more often used in T2D patients. However, after propensity score matching groups were equal to all risk factors of interest that could have influenced our cardiovascular outcomes of interest including death in the long-term follow up. The outcomes of interest in our study are of high external validity [11] and by using the NDR we can be confident that the classification of T2D was correct This is in contrast with other observational studies [20, 22], in which the discrimination between different types of diabetes was not accurate.

### Carotid intervention and diabetes

Our study showed almost a 30% relative long-term increased risk of stroke in T2D patients compared to patients without T2D after carotid intervention. Covariates involved in the associations of the outcome of interest at follow-up were all well balanced due to the propensity score matching and therefore cannot explain the higher risk of stroke and death observed in patients with T2D. The net benefit from carotid intervention depends on the complication rate as well as the risk of stroke during follow up, theoretically patients with T2D may even benefit more (relatively) if undergoing carotid intervention than patients without T2D. In the present study, the cumulative risk of stroke during 5-years follow-up in the present study was for T2D patients 16% vs. 12% in non-T2D patients. This could be compared to the 5 years cumulative risk of any stroke event of 24% in the surgical arm of the NASCET trial [21]. This demonstrates that patients undergoing carotid intervention due to a severe symptomatic carotid stenosis are at high risk for long-term complications such as stroke and death. Despite this, the benefit of CEA in patients with symptomatic carotid stenosis is durable and convincing in comparison with medical treatment alone [22].

CEA has been shown to reduce the risk of stroke in patients with symptomatic carotid stenosis [21, 23–25], whereas carotid artery stenting has evolved as an alternative minimal invasive treatment. Several randomised controlled trials have shown that CAS is associated with a higher proportion of perioperative stroke or death as compared to CEA [26], therefore the net benefit from the carotid intervention may also depend on the procedure chosen. Moreover, carotid surgery is also proven beneficial in patients with asymptomatic carotid stenosis [23, 27]. Even though the present observational study was large, the subgroups of patients undergoing CEA or CAS for symptomatic or asymptomatic stenosis were small and therefore results in these are less reliable.

### Strengths and limitations

The strengths of the present observational nationwide study were its size and the high external validity of the registries used for the outcomes of interest. The Cause of Death register used to retrieve mortality data has complete coverage of the country, and therefore we had no loss to follow-up. We used propensity score matching thereby limiting confounding factors for the results. However, our study has limitations. There is a lack of information about the type, severity, and laterality of the stroke. Also, there is a lack of information about metabolic cardiovascular risk factors such as blood pressure, blood lipids, and kidney function, i.e., estimated glomerular filtration rate which might have affected our results.

It is however known that patients with diabetes are more exposed to these cardiovascular risk factors compared to patients without diabetes. Even though the comparison of the groups was made before and after propensity score matching, thereby limiting the influence of confounding factors on the results, we cannot entirely rule out some unmeasured differences between the groups which may have affected the results.

## Conclusions

This nationwide, propensity score matched, cohort study shows that patients with T2D have an increased long-term risk for stroke and death and MACE, compared to patients without T2D, after carotid intervention. There was also an increased risk of preoperative stroke, but not death, in T2D patients compared to patients without T2D. Even though the periprocedural complications after carotid intervention today is low and acceptable, our finding highlights the importance to define certain risk groups such as diabetes.

## Supplementary Information

**Additional file 1: Table S1.** List of baseline variables adjusted for in the inverse probability of treatment weighting (IPTW) adjusted Cox regression. **Table S2.** Baseline characteristics of individuals (non-type 2 diabetes [Non-T2D] vs. type 2 diabetes [T2D]) after adjustments using inverse probability of treatment weighting [IPTW]). **Table S3.** Cox-regression estimates after adjustments using inverse probability of treatment weighting (IPTW) for individuals with type 2 diabetes compared with individuals without type 2 diabetes with asymptomatic (*n* = 147) and symptomatic (*n* = 1194) carotid stenosis undergoing any carotid intervention. **Table S4.** Cox-regression estimates after adjustments using inverse probability of treatment weighting (IPTW) for individuals with type 2 diabetes compared with individuals without type 2 diabetes with carotid endarterectomy (CEA, *n* = 1269) and carotid artery stenting (CAS, *n* = 72) procedure, respectively.

## Data Availability

The datasets analysed during the current study are available from the corresponding author on reasonable request.

## Abbreviations

BMI: Body mass index
CAS: Carotid artery stenting
CEA: Carotid endarterectomy
CI: Confidence interval
CHD: Coronary heart disease
DPP4i: Dipeptidyl peptidase 4 inhibitors
ECTS: European carotid surgery trial
eGDR: Estimated glucose disposal rate
GLP-1: Glucagon-like peptide-1
HbA1c: Glycosylated haemoglobin A1c
HR: Hazard ratio
ICD: International Classification of Disease
IPR: In patient registry
IPTW: Inverse probability of treatment weighting
IQR: Interquartile range
LISA: Longitudinal integration database for health insurance and job market studies register
MACE: Major adverse cardiac event
MI: Myocardial infarction
NASCET: North American symptomatic carotid endarterectomy trial
NDR: National diabetes register
NPR: Swedish national patient register
OR: Odds rate
PDR: Swedish prescribed drug register
SWEDVASC: Swedish national registry for vascular surgery
SD: Standard deviation
SLGT2i: Sodium glucose transporter-2 inhibitors
T2D: Type 2 diabetes
