# Supplementary material for: Early and long-term prognosis in patients with and without type 2 diabetes after carotid intervention: a Swedish nationwide propensity score matched cohort study
Source: Cardiovasc Diabetol. 2021 Apr 24;20:85. doi: 10.1186/s12933-021-01282-x (PMC8070321; doi:10.1186/s12933-021-01282-x)
Supplement: Supplementary file 1 — Additional file 1: Table S1. List of baseline variables adjusted for in the inverse probability of treatment weighting (IPTW) adjusted Cox regression. Table S2. Baseline characteristics of individuals (non-type 2 diabetes [Non-T2D] vs. type 2 diabetes [T2D]) after adjustments using inverse probability of treatment weighting [IPTW]). Table S3. Cox-regression estimates after adjustments using inverse probability of treatment weighting (IPTW) for individuals with type 2 diabetes compared with individuals without type 2 diabetes with asymptomatic (n = 147) and symptomatic (n = 1194) carotid stenosis undergoing any carotid intervention. Table S4. Cox-regression estimates after adjustments using inverse probability of treatment weighting (IPTW) for individuals with type 2 diabetes compared with individuals without type 2 diabetes with carotid endarterectomy (CEA, n = 1269) and carotid artery stenting (CAS, n = 72) procedure, respectively. [file 12933_2021_1282_MOESM1_ESM.docx]

**Additional file 1**

**Early and long-term prognosis in patients with and without type 2 diabetes after carotid surgery: a Swedish nationwide propensity score matched cohort study**

**Alexander Zabala^1^, Anders Gottsäter^2,3^, Marcus Lind^4,5^, Ann-Marie Svensson^6,7^, Björn Eliasson^7^, Rebecka Bertilsson^6^, Jan Ekelund^6^, Thomas Nyström^1*^ and Magnus Jonsson^8*^**

^1^Department of Clinical Science and Education, Karolinska Institutet, Södersjukhuset, Stockholm, Sweden

^2^Department of Clinical Sciences, Malmö, Lund University, Sweden

^3^Vascular Center, Department of Cardio Thoracic Surgery and Vascular Diseases, Skåne University Hospital, Sweden

^4^Department of Molecular and Clinical Medicine, Institute of Medicine, University of Gothenburg, Gothenburg, Sweden

^5^Department of Medicine, NU Hospital Group, Uddevalla, Sweden

^6^Centre of Registers in Region Västra Götaland, Sweden

^7^Institute of Medicine, University of Gothenburg, Gothenburg, Sweden

^8^ Department of Molecular Medicine and Surgery, Karolinska Institutet, Stockholm, Sweden.

^9^ Department of Vascular Surgery, Karolinska University Hospital, Stockholm, Sweden

**Table S1.** List of baseline variables adjusted for in the inverse probability of treatment weighting (IPTW) adjusted Cox regression.

| Age |
| --- |
| Sex |
| Smoking |
| Indication (symptomatic/asymptomatic) |
| Type of surgery (CEA/CAS) |
| Lipid lowering treatment |
| ACE inhibitor |
| Angiotensin II receptor blocker |
| Beta blocker |
| Calcium Chanel blocker |
| Anticoagulant therapy |
| Acetylsalicylic acid |
| P2Y12 inhibitor (Clopidogrel) |
| Disposable income |
| Education |
| Marital status |
| Country of origin |
| Cardiovascular disease |
| Stroke |
| Myocardial infarction |
| Coronary heart disease |
| Heart failure |
| Atrial fibrillation |
| Kidney disease |
| Cancer disease |
| Gastric bypass |
| Psychiatric disorder |
| Dementia |

**Table S2.** Baseline characteristics of individuals (non-type 2 diabetes [Non-T2D] vs. type 2 diabetes [T2D]) after adjustments using inverse probability of treatment weighting [IPTW]).

|  | Non-T2D | T2D | P-value | SMD |
| --- | --- | --- | --- | --- |
| n | 5400.61 | 4709.6 |  |  |
| Age, mean (SD) | 72.28 (8.16) | 72.33 (7.72) | 0.849 | 0.007 |
| Female, n (%) | 1727.5 (32.0) | 1399.7 (29.7) | 0.170 | 0.049 |
| Smoking, n (%) | 1000.2 (24.3) | 963.7 (23.5) | 0.632 | 0.019 |
| Medication, n (%) |  |  |  |  |
| Lipid lowering drug | 3589.3 (66.5) | 3264.6 (69.3) | 0.108 | 0.061 |
| Antihypertensive drug | 4309.4 (79.8) | 3876.2 (82.3) | 0.138 | 0.064 |
| ACE inhibitors | 1630.6 (30.2) | 1541.0 (39.7) | 0.120 | 0.055 |
| Angiotensin II receptor blocker | 1048.8 (19.4) | 980.5 (20.8) | 0.282 | 0.035 |
| Beta blocker | 2444.4 (45.3) | 2232.2 (47.4) | 0.218 | 0.043 |
| Calcium channel blocker | 1956.4 (36.2) | 1815.3 (38.5) | 0.155 | 0.048 |
| Anticoagulant therapy^a^ | 1945.8 (36.0) | 1709.1 (36.3) | 0.873 | 0.005 |
| Acetylsalicylic acid | 3351.7 (62.1) | 2991.6 (63.5) | 0.400 | 0.030 |
| P2Y12 inhibitor (Clopidogrel) | 1021.5 (18.9) | 896.3 (19.0) | 0.003 | 0.092 |
| Antihyperglycaemic agent, n (%) |  |  |  |  |
| Metformin | 0 (0.0) | 744 (55.5) | <0.001 | 1.511 |
| Sodium-glucose-transport-2 inhibitor | 0.0 ( 0.0) | 15.4 ( 0.3) | 0.031 | 0.081 |
| Incretin^b^ | 0.0 ( 0.0) | 258.5 ( 5.5) | <0.001 | 0.341 |
| Insulin | 0.0 ( 0.0) | 1539.2 (32.7) | <0.001 | 0.985 |
| Disposable income per month after tax, USD | 2024.55 (2468.21) | 1928.45 (1706.19) | 0.100 | 0.045 |
| Educational level, n (%) |  |  | 0.267 | 0.058 |
| Compulsory school | 2169.4 (40.6) | 1866.9 (40.0) |  |  |
| Upper secondary | 2233.8 (41.8) | 2061.1 (44.2) |  |  |
| College/University | 940.6 (17.6) | 736.2 (15.8) |  |  |
| Civil status, n (%) |  |  | 0.887 | 0.028 |
| Single | 507.3 (9.4) | 443.5 (9.4) |  |  |
| Married | 2967.0 (55.0) | 2548.6 (54.2) |  |  |
| Divorced | 1089.0 (20.2) | 1000.9 (21.3) |  |  |
| Widowed | 832.6 (15.4) | 708.7 (15.1) |  |  |
| Origin, n (%) |  |  | 0.886 | 0.016 |
| Sweden | 4607.1 (85.3) | 3995.4 (84.8) |  |  |
| Europe except Sweden | 399.2 (7.4) | 350.2 (7.4) |  |  |
| Rest of the world | 394.3 (7.3) | 363.6 (7.7) |  |  |
| History of comorbidities, n (%) |  |  |  |  |
| Cardiovascular disease (%) | 3045.5 (56.4) | 2757.4 (58.6) | 0.218 | 0.044 |
| Stroke | 2663.2 (49.3) | 2442.9 (51.9) | 0.146 | 0.051 |
| Myocardial infarction | 708.3 (13.1) | 652.5 (13.9) | 0.495 | 0.022 |
| Coronary heart disease | 1608.6 (29.8) | 1516.5 (32.2) | 0.117 | 0.052 |
| Heart failure | 357.9 (6.6) | 332.8 (7.1) | 0.562 | 0.017 |
| Atrial fibrillation | 644.6 (11.9) | 583.1 (12.4) | 0.655 | 0.014 |
| Kidney disease | 220.6 (4.1) | 226.9 (4.8) | 0.276 | 0.036 |
| Cancer disease | 585.1 (10.8) | 495.6 (10.5) | 0.779 | 0.010 |
| Gastric bypass | 2.5 (0.0) | 3.7 (0.1) | 0.611 | 0.012 |
| Psychiatric disorder | 198.8 (3.7) | 170.0 (3.6) | 0.272 | 0.038 |
| Dementia | 20.5 (0.4) | 13.5 (0.3) | 0.592 | 0.016 |
| Degree of Ipsilateral Carotid Stenosis, n (%)^c^ |  |  | 0.269 | 0.057 |
| ≤50% | 289.7 (5.4) | 291.3 (6.2) |  |  |
| 50-69% | 1551.5 (28.7) | 1437.0 (30.5) |  |  |
| 70-99% | 3557.2 (65.9) | 2980.9 (63.3) |  |  |
| Degree of Contralateral Carotid Stenosis, n (%)^c^ |  |  | 0.541 | 0.050 |
| ≤50% | 4002.3 (74.2) | 3435.2 (72.9) |  |  |
| 50-69% | 594.6 (11.0) | 562.3 (11.9) |  |  |
| 70-99% | 461.9 (8.6) | 446.0 (9.5) |  |  |
| Occlusion | 338.5 (6.3) | 265.7 (5.6) |  |  |
| Symptomatic stenosis, n (%) | 4782.7 (88.6) | 4197.1 (89.1) | 0.597 | 0.018 |
| Carotid endarterectomy, n (%) | 5176.3 (95.8) | 4512.6 (95.8) | 0.973 | 0.062 |

^*^Anticoagulant therapy includes Heparin, Low molecular Heparin, Non-Vitamin K antagonist and Vitamin K antagonists. ^**^Incretin includes dipeptidyl peptidase-4 inhibitors and glucagon-like peptide-1. ^***^Definition accordingly to The North American Symptomatic Carotid Endarterectomy Trial. SMD, Standardised mean difference. SD, Standard deviation. Categorical variables are presented as number (%) and

continuous variables are presented as mean (SD).

**Table S3.** Cox-regression estimates after adjustments using inverse probability of treatment weighting [IPTW]) for individuals with type 2 diabetes compared with individuals without type 2 diabetes with asymptomatic (*n*=147) and symptomatic (*n*=1194) carotid stenosis undergoing any carotid intervention.

| Outcome | **Proportion**  **of events**  **(Symptomatic)**  **(%)** | **Symptomatic carotid stenosis**  **HR (95% CI)** | **P-value** | **Proportion**  **of events**  **(Asymptomatic)**  **(%)** | **Asymptomatic carotid stenosis**  **HR (95% CI)** | **P-value** |
| --- | --- | --- | --- | --- | --- | --- |
| Stroke | 91.3 | 1.32 (1.09 - 1.60) | 0.005 | 8.7 | 0.88 (0.40-0.90) | 0.741 |
| Ischemic stroke | 92.1 | 1.33 (1.08 - 1.64) | 0.008 | 7.9 | 1.06 (0.46-2.44) | 0.892 |
| Hemorrhagic stroke | 84.2 | 1.34 (1.10 - 1.63) | 0.030 | 15.8 | 0.83 (0.39-1.76) | 0.623 |
| Death | 90.8 | 1.29 (1.11 - 1.50) | 0.001 | 9.2 | 1.19 (0.72-1.97) | 0.503 |
| Cardiovascular death | 93.6 | 1.64 (1.22 - 2.19) | 0.009 | 6.4 | 1.33 (0.44-4.02) | 0.609 |
| MACE | 88.3 | 1.21 (1.08 - 1.64) | 0.002 | 11.7 | 1.28 (0.90-1.82) | 0.170 |

CI, Confidence Interval; HR, Hazard Ratio

**Table S4.** Cox-regression estimates after adjustments using inverse probability of treatment weighting [IPTW]) for individuals with type 2 diabetes compared with individuals without type 2 diabetes with carotid endarterectomy (CEA, *n*=1269) and carotid artery stenting (CAS, *n*=72) procedure, respectively.

| Outcome | **Proportion**  **of events**  **(CEA)**  **(%)** | **CEA**  **HR (95% CI)** | **P-value** | **Proportion of events**  **(CAS)**  **(%)** | **CAS**  **HR (95% CI)** | **P-value** |
| --- | --- | --- | --- | --- | --- | --- |
| Stroke | 93.2 | 1.29 (1.06 - 1.56) | 0.011 | 6.8 | 1.47 (0.76 - 2.84) | 0.247 |
| Ischemic stroke | 92.0 | 1.3 (1.05 - 1.61) | 0.001 | 8.0 | 1.80 (0.90-3.63) | 0.098 |
| Hemorrhagic stroke | 95.2 | 1.32 (1.09 - 1.61) | 0.005 | 4.8 | 1.06 (0.56 - 1.99) | 0.859 |
| Death | 93.3 | 1.29 (1.11 - 1.51) | 0.008 | 6.7 | 1.18 (0.70 – 1.96) | 0.541 |
| Cardiovascular death | 92.7 | 1.54 (1.14 - 2.07) | 0.005 | 7.3 | 2.49 (0.93 - 6.64) | 0.070 |
| MACE | 94.0 | 1.22 (1.08 - 1.37) | 0.001 | 6.0 | 1.54 (1.00 - 2.37) | 0.050 |

CI, Confidence Interval; HR, Hazard Ratio

**The RECORD statement – checklist of items, extended from the STROBE statement, that should be reported in observational studies using routinely collected health data.**

|  | **Item No.** | **STROBE items** | **Location in manuscript where items are reported** | **RECORD items** | **Location in manuscript where items are reported** |
| --- | --- | --- | --- | --- | --- |
| **Title and abstract** | | | | | |
|  | 1 | (a) Indicate the study’s design with a commonly used term in the title or the abstract (b) Provide in the abstract an informative and balanced summary of what was done and what was found | Page 1-4 | RECORD 1.1: The type of data used should be specified in the title or abstract. When possible, the name of the databases used should be included.  RECORD 1.2: If applicable, the geographic region and timeframe within which the study took place should be reported in the title or abstract.  RECORD 1.3: If linkage between databases was conducted for the study, this should be clearly stated in the title or abstract. |  |
| **Introduction** | | | | | |
| Background rationale | 2 | Explain the scientific background and rationale for the investigation being reported | Page 5 |  |  |
| Objectives | 3 | State specific objectives, including any prespecified hypotheses | Page 5 |  |  |
| **Methods** | | | | | |
| Study Design | 4 | Present key elements of study design early in the paper | Page 6 |  |  |
| Setting | 5 | Describe the setting, locations, and relevant dates, including periods of recruitment, exposure, follow-up, and data collection | Page 6-8 |  |  |
| Participants | 6 | *(a) Cohort study* - Give the eligibility criteria, and the sources and methods of selection of participants. Describe methods of follow-up  *Case-control study* - Give the eligibility criteria, and the sources and methods of case ascertainment and control selection. Give the rationale for the choice of cases and controls  *Cross-sectional study* - Give the eligibility criteria, and the sources and methods of selection of participants  *(b) Cohort study* - For matched studies, give matching criteria and number of exposed and unexposed  *Case-control study* - For matched studies, give matching criteria and the number of controls per case | a;Page 6-7  b;Page | RECORD 6.1: The methods of study population selection (such as codes or algorithms used to identify subjects) should be listed in detail. If this is not possible, an explanation should be provided.  RECORD 6.2: Any validation studies of the codes or algorithms used to select the population should be referenced. If validation was conducted for this study and not published elsewhere, detailed methods and results should be provided.  RECORD 6.3: If the study involved linkage of databases, consider use of a flow diagram or other graphical display to demonstrate the data linkage process, including the number of individuals with linked data at each stage. |  |
| Variables | 7 | Clearly define all outcomes, exposures, predictors, potential confounders, and effect modifiers. Give diagnostic criteria, if applicable. | Page 6-8 | RECORD 7.1: A complete list of codes and algorithms used to classify exposures, outcomes, confounders, and effect modifiers should be provided. If these cannot be reported, an explanation should be provided. |  |
| Data sources/ measurement | 8 | For each variable of interest, give sources of data and details of methods of assessment (measurement).  Describe comparability of assessment methods if there is more than one group | Page 6-7 |  |  |
| Bias | 9 | Describe any efforts to address potential sources of bias | Page 9 |  |  |
| Study size | 10 | Explain how the study size was arrived at | Page 7 |  |  |
| Quantitative variables | 11 | Explain how quantitative variables were handled in the analyses. If applicable, describe which groupings were chosen, and why | Page 9-10 |  |  |
| Statistical methods | 12 | (a) Describe all statistical methods, including those used to control for confounding  (b) Describe any methods used to examine subgroups and interactions  (c) Explain how missing data were addressed  (d) *Cohort study* - If applicable, explain how loss to follow-up was addressed  *Case-control study* - If applicable, explain how matching of cases and controls was addressed  *Cross-sectional study* - If applicable, describe analytical methods taking account of sampling strategy  (e) Describe any sensitivity analyses | Page 9-10 |  |  |
| Data access and cleaning methods |  | .. | Page 6-7 | RECORD 12.1: Authors should describe the extent to which the investigators had access to the database population used to create the study population.  RECORD 12.2: Authors should provide information on the data cleaning methods used in the study. |  |
| Linkage |  | .. | Page 6 | RECORD 12.3: State whether the study included person-level, institutional-level, or other data linkage across two or more databases. The methods of linkage and methods of linkage quality evaluation should be provided. |  |
| **Results** | | | | | |
| Participants | 13 | (a) Report the numbers of individuals at each stage of the study (*e.g.*, numbers potentially eligible, examined for eligibility, confirmed eligible, included in the study, completing follow-up, and analysed)  (b) Give reasons for non-participation at each stage.  (c) Consider use of a flow diagram | a) Page 10 | RECORD 13.1: Describe in detail the selection of the persons included in the study (*i.e.,* study population selection) including filtering based on data quality, data availability and linkage. The selection of included persons can be described in the text and/or by means of the study flow diagram. |  |
| Descriptive data | 14 | (a) Give characteristics of study participants (*e.g.*, demographic, clinical, social) and information on exposures and potential confounders  (b) Indicate the number of participants with missing data for each variable of interest  (c) *Cohort study* - summarise follow-up time (*e.g.*, average and total amount) | a) Page 10 and Table 1  b) Page 10  c) Page 11 |  |  |
| Outcome data | 15 | *Cohort study* - Report numbers of outcome events or summary measures over time  *Case-control study* - Report numbers in each exposure category, or summary measures of exposure  *Cross-sectional study* - Report numbers of outcome events or summary measures | Page 10 and table 2. |  |  |
| Main results | 16 | (a) Give unadjusted estimates and, if applicable, confounder-adjusted estimates and their precision (e.g., 95% confidence interval). Make clear which confounders were adjusted for and why they were included  (b) Report category boundaries when continuous variables were categorized  (c) If relevant, consider translating estimates of relative risk into absolute risk for a meaningful time period | a) Table 2,3  Figure A-D  b) Page 7 |  |  |
| Other analyses | 17 | Report other analyses done—e.g., analyses of subgroups and interactions, and sensitivity analyses | N/A |  |  |
| **Discussion** | | | | | |
| Key results | 18 | Summarise key results with reference to study objectives | Page 12-15 |  |  |
| Limitations | 19 | Discuss limitations of the study, taking into account sources of potential bias or imprecision. Discuss both direction and magnitude of any potential bias | Page 15 | RECORD 19.1: Discuss the implications of using data that were not created or collected to answer the specific research question(s). Include discussion of misclassification bias, unmeasured confounding, missing data, and changing eligibility over time, as they pertain to the study being reported. |  |
| Interpretation | 20 | Give a cautious overall interpretation of results considering objectives, limitations, multiplicity of analyses, results from similar studies, and other relevant evidence | Page 16 |  |  |
| Generalisability | 21 | Discuss the generalisability (external validity) of the study results | Page 12-15 |  |  |
| **Other Information** | | | | | |
| Funding | 22 | Give the source of funding and the role of the funders for the present study and, if applicable, for the original study on which the present article is based | Page 16 |  |  |
| Accessibility of protocol, raw data, and programming code |  | .. | Page 17 | RECORD 22.1: Authors should provide information on how to access any supplemental information such as the study protocol, raw data, or programming code. |  |

*Reference: Benchimol EI, Smeeth L, Guttmann A, Harron K, Moher D, Petersen I, Sørensen HT, von Elm E, Langan SM, the RECORD Working Committee. The REporting of studies Conducted using Observational Routinely-collected health Data (RECORD) Statement. *PLoS Medicine* 2015;12(10):e1001885. DOI: 10.1371/journal.pmed.1001885. *Checklist is protected under Creative Commons Attribution ([CC BY](http://creativecommons.org/licenses/by/4.0/)) license.
